# Diversity and Versatility of Phagocytosis: Roles in Innate Immunity, Tissue Remodeling, and Homeostasis

**DOI:** 10.3389/fcimb.2017.00191

**Published:** 2017-05-23

**Authors:** Justin J. Lim, Sergio Grinstein, Ziv Roth

**Affiliations:** ^1^Program in Cell Biology, Hospital for Sick ChildrenToronto, ON, Canada; ^2^Keenan Research Centre for Biomedical Science, St. Michael's HospitalToronto, ON, Canada; ^3^Department of Biochemistry, University of TorontoToronto, ON, Canada

**Keywords:** phagocytosis, phagocyte, phagosome, neutrophil, macrophage

## Abstract

Phagocytosis, a critical early event in the microbicidal response of neutrophils, is now appreciated to serve multiple functions in a variety of cell types. Professional phagocytes play a central role in innate immunity by eliminating pathogenic bacteria, fungi and malignant cells, and contribute to adaptive immunity by presenting antigens to lymphocytes. In addition, phagocytes play a part in tissue remodeling and maintain overall homeostasis by disposing of apoptotic cells, a task shared by non-professional phagocytes, often of epithelial origin. This functional versatility is supported by a vast array of receptors capable of recognizing a striking variety of foreign and endogenous ligands. Here we present an abbreviated overview of the different types of phagocytes, their varied modes of signaling and particle engulfment, and the multiple physiological roles of phagocytosis.

## Introduction

Phagocytosis is an important component of the microbicidal function of neutrophils; however, phagocytosis is also utilized by a variety of cell types in several physiological contexts. Elie Metchnikoff, commonly referred to as the father of innate immunity, explored phagocytosis in polymorphonuclear neutrophils, and macrophages (Gordon, 2008). Since then, many more metazoan cells have been described to display phagocytic capacity. The main objective of this review is to provide an overview of the multiplicity of functions served by phagocytes.

Phagocytic cells can be organized into two categories: the professional and non-professional phagocytes (Rabinovitch, 1995). Professional or dedicated phagocytes consist primarily of polymorphonuclear neutrophils, monocytes, monocyte-derived macrophages, and tissue-resident macrophages—cells whose raison d'être is phagocytosis. Non-professional phagocytes include all other cell types that can perform phagocytosis, such as epithelial cells, fibroblasts, and dendritic cells (DCs). Such cells have a more restricted set of targets and engulf them more slowly. Thus, the phagocytic capacity of non-professional phagocytes differs significantly in scope and efficiency from that of professional phagocytes. Both professional and non-professional phagocytes play important roles in innate immunity due to their ability to engulf pathogens. However, they are also vital in the maintenance of the healthy physiological state, in recovery from injury and in the development of the host organism (Mallat et al., 2005; Arandjelovic and Ravichandran, 2015; Wynn and Vannella, 2016). Thus, the consequences of phagocytosis extend well beyond the context of innate immunity.

As a form of receptor-mediated endocytosis, phagocytosis involves a variety of receptors, which are essential for the recognition and internalization of multiple ligands, reflecting its diverse functions (Gordon, 2016). Nevertheless, the gross phenotype of phagocytosis is relatively constant. It begins with the recognition of the target, i.e., the binding of a phagocytic receptor to its cognate ligand. Foreign particles and altered self cells are the two main targets of phagocytosis (Gordon, 2016). The term “altered self” typically refers to apoptotic and necrotic cells; however, it is now recognized that viable-but-stressed cells can also be internalized by a process labeled as phagoptosis (Brown and Neher, 2012). Self (host) cells generally express “don't-eat-me” signals, such as CD47, and its receptor on myeloid cells, SIRPα, inhibits phagocytosis (Hochreiter-Hufford and Ravichandran, 2013). These signals are down-regulated in altered self cells and “eat-me-signals,” such as phosphatidylserine (PS), become exposed at the cell surface. Receptors that recognize eat-me-signals involve those that bind directly to PS or PS-binding bridging proteins (named collectively as PS receptors), altered sugars (recognized by lectins), and thrombospondin (Hochreiter-Hufford and Ravichandran, 2013). Receptors involved in the phagocytosis of foreign particles include those that bind to opsonins (e.g., Fc and complement receptors), pathogen-associated molecular patterns (recognized by pattern-recognition receptors), and scavenger receptors (Freeman and Grinstein, 2014). Binding of multivalent ligand on the surface of the target particle results in receptor clustering and, after several intervening steps, in the recruitment of Rho family GTPases (Tollis et al., 2010). Subsequent signaling leads to the actin-dependent formation of the phagocytic cup and the extension of pseudopodia around the ligand, culminating in internalization. The target is taken into a vacuole—the phagosome—which undergoes extensive remodeling, a process known as maturation, that renders it hostile to microbes and well suited for the degradation of the internalized particle (Kinchen and Ravichandran, 2008). This general pattern applies to virtually all targets, although substantive differences exist depending on the phagocyte and target engaged.

The wide variety of phagocytic targets that require internalization and elimination mirrors the diverse functions of phagocytosis. This review describes several examples of the process as it applies to tissue homeostasis and remodeling, and also in the context of innate immunity, in an attempt to convey the multiplicity of phagocytic functions and the versatile nature of the phagocytes themselves. In each case, we endeavored to describe the physiological outcomes of phagocytosis and explore at the molecular level the cascade of events initiated by receptor-ligand interactions.

## Resolution of inflammation

Cells of the innate immune system play a central role in acute inflammation. In particular, neutrophils, and macrophages are critical in the response to perturbations of tissue homeostasis. Neutrophils are typically the first to arrive at sites of injury and they are responsible for the removal of the pathogens or pro-inflammatory stimuli (Butterfield et al., 2006; Koh and DiPietro, 2011; Kolaczkowska and Kubes, 2013) (their phagocytic role in acute inflammation is discussed below). Monocyte-derived macrophages and tissue resident macrophages also participate in the onset of the inflammatory event, but, in addition, they are crucial to the development of subsequent events, specifically, the resolution of inflammation.

Non-phlogistic elimination of activated immune cells at the site of injury is essential to the resolution of inflammation. The removal of spent neutrophils is a prominent feature of this process, as dying cells can release histotoxic molecules that would prolong inflammation. Indeed, impaired removal of neutrophils has been associated with various chronic inflammatory diseases, such as HIV and lupus (Torre et al., 2002; Potter et al., 2003). Early studies of the resolution of inflammation speculated that macrophages mediate the elimination of neutrophils from the inflammatory site. Macrophages were shown to have engulfed neutrophils at inflammatory lesions *in vivo;* moreover, activated neutrophils have not been observed to leave the damaged area (Savill et al., 1989; Haslett, 1992; Cox et al., 1995).

**Figure 1:**
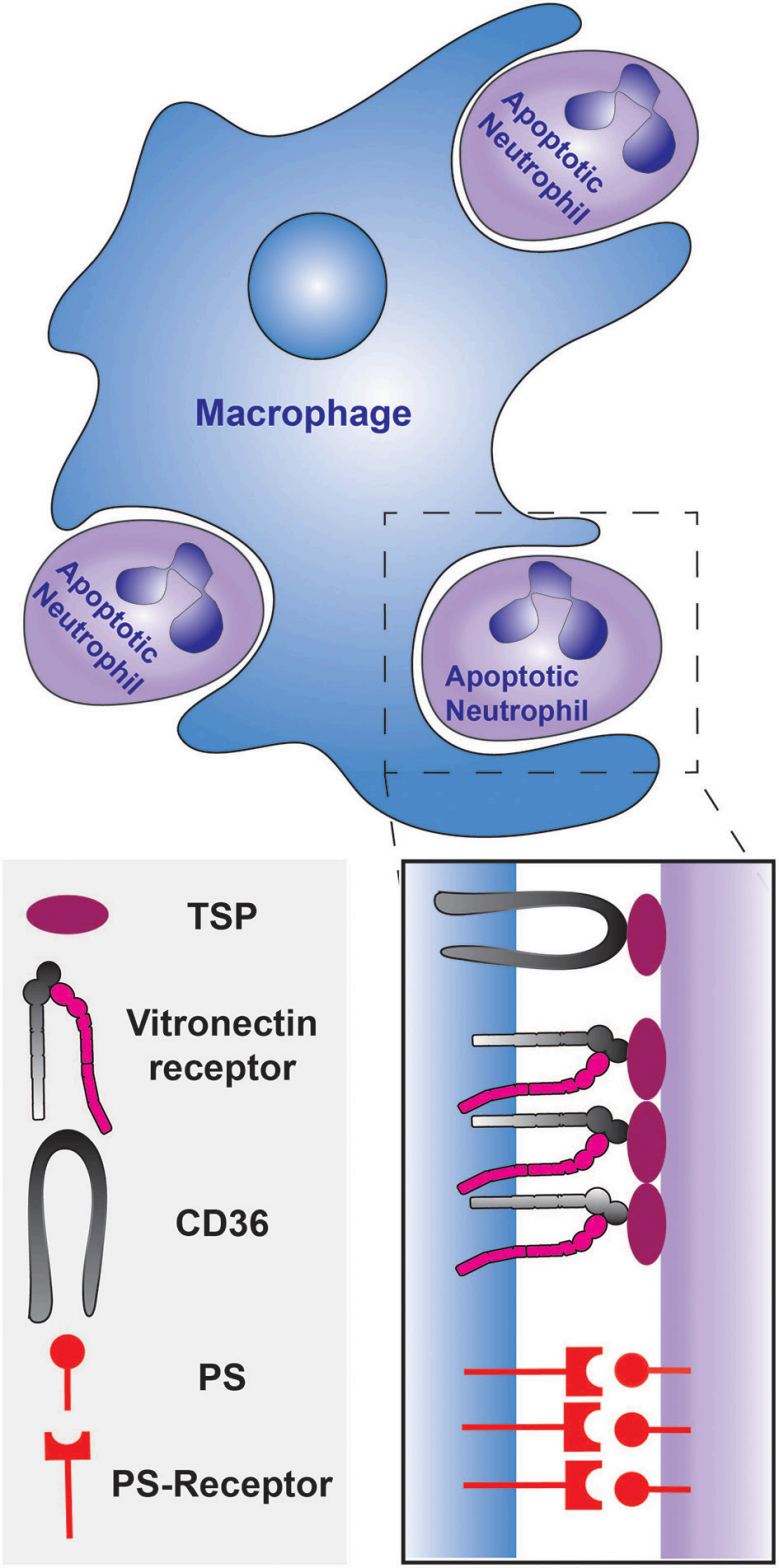
**Phagocytosis of apoptotic neutrophils by a macrophage during the resolution of inflammation**. The engulfment can be mediated by PS and/or the opsonization of the apoptotic neutrophils by thrombospondin. The thrombospondin-coated apoptotic cells are tethered to the macrophage by CD36, and the vitronectin receptor signals the initiation of phagocytosis. PS is recognized by the PS-receptor on the macrophage.

Subsequent studies that investigated the mechanism of uptake found that elimination is triggered by neutrophil apoptosis. Isolated neutrophils from human peripheral blood were shown to undergo apoptosis within 24 h of plating *in vitro* and the fraction of apoptotic neutrophils positively correlated with their recognition and ingestion by macrophages (Savill et al., 1989). This occurrence was validated *in vivo* by numerous histological studies and by analyses of broncho-alveolar lavages (Haslett et al., 1994; Cox et al., 1995; Ishii et al., 1998). Although apoptotic cells are primarily recognized via PS receptors, the engulfment of dying neutrophils was discovered to be largely dependent on the integrin receptor for vitronectin (Savill et al., 1990; Fadok et al., 1998). PS-mediated engulfment becomes significant only upon the down-regulation of the vitronectin receptor, which can be accomplished by prolonged stimulation with β-1,3 glucan (Fadok et al., 1998). As depicted in Figure 1, the target ligand of the vitronectin receptor was found to be thrombospondin, that acts as a molecular bridge to the apoptotic neutrophil by engaging PS on the apoptotic cell surface (Savill et al., 1992; Gayen Betal and Setty, 2008). In addition, CD36 was also found to bind thrombospondin to tether the macrophage against the neutrophil cell surface, facilitating phagocytosis (Savill et al., 1992; Fadok et al., 1998). The LRP1 receptor, which binds to calreticulin on apoptotic cells, has also been shown to contribute to the phagocytosis of apoptotic neutrophils (Gabillet et al., 2012). Clearly, removal of apoptotic cells is a complex, multifactorial phenomenon; several receptors and mechanisms are likely to serve concomitant roles. The origin and polarization state of the macrophages may introduce additional complexity (Visser et al., 1995).

## Red cell biogenesis and elimination

The biogenesis and elimination of erythrocytes is closely tied to phagocytosis. Because of their relatively short lifespan (≈120 days), erythrocytes must be constantly produced (at a rate of ≈2 million cells per second in humans). Maintenance of homeostasis requires ongoing clearance of effete cells, a process undertaken by macrophages. As a result, modulation of the erythrocyte life cycle is one of the most prominent functions of phagocytosis (Brown and Neher, 2012; Dzierzak and Philipsen, 2013).

Erythropoiesis within the adult mammal involves the step-wise differentiation of pluripotent hematopoietic stem cells within the bone marrow to megakaryocyte-erythroid progenitor cells (Psaila et al., 2016). These progenitor cells then direct their differentiation to produce either platelets or mature red blood cells (RBCs) (de Back et al., 2014; Psaila et al., 2016). An important step in the erythropoietic pathway is the expulsion of the nucleus from the committed erythroblast, to produce reticulocytes and mature RBCs (de Back et al., 2014; Psaila et al., 2016). The first conclusive evidence of enucleation via physical expulsion of the nucleus was provided by electron micrographs of hematopoiesis in fetal guinea pig livers (Campbell, 1968). Such images showed processes extending from macrophages that surrounded the nuclei being extruded, which explains the absence of free extracellular nuclei at sites of hematopoiesis (Skutelsky and Danon, 1969). Engulfment of expelled nuclei by macrophages was also recorded at other hematopoietic sites, such as the spleen and bone marrow (Manwani and Bieker, 2008). Consistent with these findings, it was known that erythroblastic islands, consisting of a central macrophage surrounded by developing erythroblasts, exist in the bone marrow (Mohandas and Prenant, 1978). These central macrophages within the islands are responsible for the engulfment of ejected nuclei (Sasaki et al., 1993a,b). The ingested nuclei must then be digested by the phago-lysosome, a process that seemingly involves DNase II. The importance of this pathway is highlighted by the abnormal erythropoiesis reported in mice lacking this nuclease; such mice are severely anemic and die at the embryonic stage, perhaps owing to the inability of macrophages to digest the engulfed nuclei without DNase II (Kawane et al., 2001).

The mechanism of phagocytic engulfment of the expelled nuclei is not fully understood. It has been suggested that phospholipid asymmetry is lost and surface charge is altered in the membrane enclosing the extruding nucleus (Skutelsky and Danon, 1969; McEvoy et al., 1986). Indeed, it has been shown that PS is exposed on the surface of expelled nuclei, and masking of PS significantly reduces the efficiency of nuclear engulfment (Yoshida et al., 2005). However, the addition of phospho-L-serine does not inhibit nuclei phagocytosis; thus the primary ligand responsible for the initiation of nuclei engulfment remains uncertain (Qiu et al., 1995). Not surprisingly, the receptors involved in the engulfment of the extruded nuclei have not been conclusively identified.

Upon enucleation and differentiation into mature RBCs, the erythrocytes are released into the blood stream. Erythrocytes age in the blood stream as they become progressively damaged in the course of performing their essential functions (de Back et al., 2014). Microvesiculation has been recognized as a major mechanism of erythrocyte senescence; it results in irreversible membrane loss, with decrease in membrane flexibility that is required for movement through narrow capillary beds (Antonelou et al., 2010). These senescent RBCs are eliminated in the liver, spleen, bone marrow, blood, and lung via phagocytic engulfment by both resident and monocyte-derived macrophages (Theurl et al., 2016). Phagocytic removal is absolutely crucial for such clearance, as an increased level of damaged (membrane-compromised) RBCs could increase the level of circulating iron to toxic levels (Hentze et al., 2010).

The mechanism whereby senescent erythrocytes are engulfed involves the down-regulation of CD47, an anti-phagocytic “don't-eat-me” signal, on the erythrocyte surface (Khandelwal et al., 2007; Olsson and Oldenborg, 2008), and the upregulation of phagocytic “eat-me” signals. The latter promotes complement-mediated and PS-mediated engulfment. At the core of the complement-mediated engulfment mechanism is the transmembrane protein, band 3. Band 3 and its naturally occurring antibody typically have a low binding affinity for each other; this safeguard prevents opsonisation of young and healthy erythrocytes (Arese et al., 2005). However, with the accumulation of oxidative damage, band 3 forms clusters that act as high-affinity binding sites for the IgG anti-band 3 antibody (Kannan et al., 1991). Despite the increased binding affinity, physiological levels of the antibody are too low to stimulate FcγR-mediated phagocytosis. Instead, phagocytosis is dependent on the activation of the classical complement system by the antibody, that triggers the C3b and iC3b opsonisation of the senescent RBC (Lutz et al., 1987, 1990). Indeed, the complement receptors CR1 and CR3 have been implicated in the uptake of senescent and diseased RBCs (Gattegno et al., 1989; Lin et al., 2015). In addition to band 3 clustering, senescent RBCs display PS on their cell membrane (Connor et al., 1994; Boas et al., 1998; Lee et al., 2011). Although the particular phagocytic receptor(s) involved have not been identified, scavenger receptors have been postulated as responsible for the PS-mediated uptake of aged RBCs (Sambrano et al., 1994; Terpstra and van Berkel, 2000).

## Synaptic remodeling

Synaptic connections between neurons establish the neural networks of the central nervous system (CNS). These intercellular connections must be carefully regulated to ensure normal development of the CNS. Synaptogenesis in the human CNS begins late in fetal life and continues until the second postnatal year (Huttenlocher and Dabholkar, 1997). Afterwards, synaptic density is decreased well into adulthood, before stabilizing (Petanjek et al., 2011). This period of activity-dependent synaptic pruning is crucial to the proper development and functionality of the CNS, as superfluous connections are removed to improve the coherence of neuronal communication. The importance of developmental synaptic pruning is emphasized by the observation of alterations in diseased states. For instance, increased synaptic densities are observed in individuals with autism spectrum disorders (Tang et al., 2014). Synaptic pruning results from a combination of several degenerative processes that include membrane fragmentation, proteasome-dependent protein turnover, and cytoskeletal degeneration (Low and Cheng, 2006). The degenerating synaptic boutons must be eliminated and glial cells are key players in this process, as shown originally in *Drosophila melanogaster* (Awasaki and Ito, 2004; Watts et al., 2004).

Microglia are the phagocytic glial cells of the mammalian CNS (Doherty et al., 2009). In the healthy brain, microglia act as dynamic sentinels, constantly sending out processes to surveil their surroundings for threats to homeostasis (Kettenmann et al., 2011). Importantly, they also function as housekeepers that support adult neurogenesis by remodeling synapses in response to disuse or ischemic injuries (Wake et al., 2009; London et al., 2013). Their phagocytic role in developmental synaptic pruning is now well established. It was unveiled in fractalkine receptor-knockout mice that displayed transient reductions in microglial populations. This decrease in phagocytes was accompanied by a temporary increase in synaptic density (Paolicelli et al., 2011). Subsequently, engulfment of synaptic fragments by microglia was directly observed in the brain of developing mice (Paolicelli et al., 2011; Schafer et al., 2012). The phagocytic activity of microglia was determined to be developmentally regulated and activity-dependent, extending the relationship between microglial phagocytosis and developmental synaptic pruning (Schafer et al., 2012).

**Figure 2:**
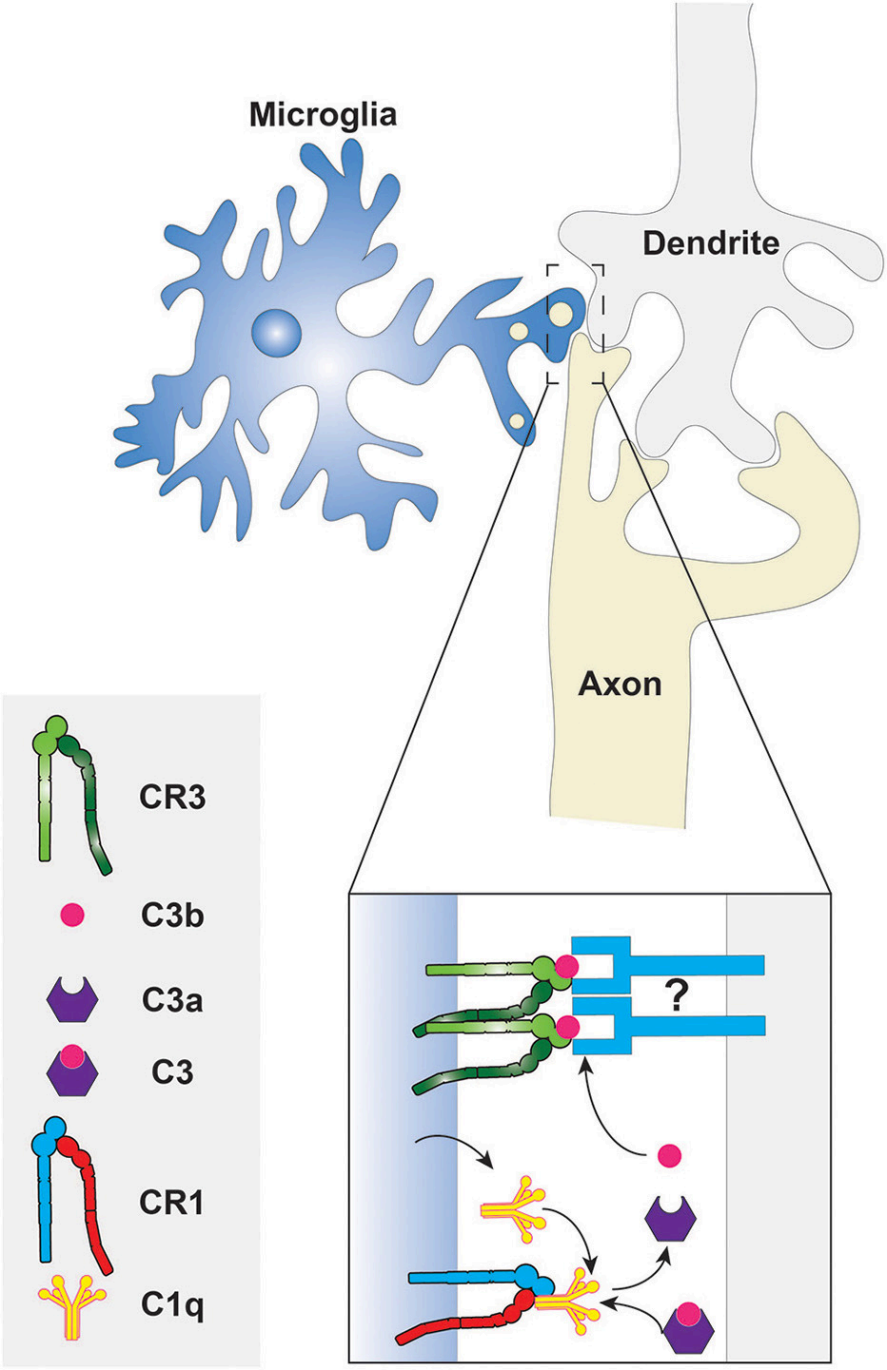
**Phagocytosis of synaptic components by microglia**. The classical complement cascade is initiated by the accumulation of C1q, produced by the microglia, at degenerating synapses. The C1q molecules bind CR1 on the microglial surface to form the C1 complex resulting in the eventual cleavage of C3. The fragments of C3 then opsonize the synaptic surface for subsequent phagocytosis via CR3. What targets C3 fragments to the synaptic membrane is unknown, thus it is depicted here as an unidentified molecule that may be a protein or a phospholipid. Other components of the C1 complex and complement cascade are not shown because they have not been studied in the context of synaptic pruning.

The molecular mechanism of phagocytic synaptic pruning has been investigated in some detail in the developing mouse (Stevens et al., 2007; Schafer et al., 2012). The complement cascade protein, C1q, was shown to be localized in regions where synaptic remodeling was underway, suggesting that the classical complement cascade is involved (Stevens et al., 2007). Indeed, C1q, C3, and CR3 were found to be necessary for proper synaptic pruning by the microglia, since knockout mice lacking these complement cascade components displayed insufficient synaptic pruning (Stevens et al., 2007; Schafer et al., 2012). Furthermore, microglia are known to express CR1 and C1q; thus it can be speculated that C3 fragments are produced in the synaptic pruning microenvironment following the formation of the C1q complex, as shown in Figure 2 (Korotzer et al., 1995; Crehan et al., 2013). The other components of the C1 complex and the complement cascade are not shown in the figure because their involvement in synaptic pruning has not been directly established. C3b then opsonizes “weak” synapses or synapses with reduced activity and phagocytosis ensues via the CR3 receptor or some C3b product-binding receptor that is expressed by microglia. Although further studies investigating the molecular mechanisms of synaptic pruning are necessary, it is evident that phagocytosis plays a key role. This provides another reminder that phagocytosis has roles not only in innate immunity and in homeostasis, but also in development.

## Adult neurogenesis

Neurogenesis, the development of neurons, was once thought to be a process exclusive to the embryonic, fetal, and postnatal stages of life; however, it is now apparent that new neurons continue to be generated in certain regions of the adult mammalian brain for the maintenance of homeostasis (Braun and Jessberger, 2014). Unexpectedly, phagocytosis was found to be required for adequate regulation of neurogenesis: clearance of apoptotic neuroprogenitors at adult neurogenic sites seems to be essential (Lu et al., 2011; Luo et al., 2016). Annexin V-mediated inhibition of apoptotic cell phagocytosis in the brain of adult mice resulted in the obstruction of neuronal differentiation (Lu et al., 2011). It remains unclear whether phagocytosis of the apoptotic corpses was carried out by neuronal precursor cells (Lu et al., 2011), or by microglia present at neurogenic sites (Sierra et al., 2010; Luo et al., 2016). Regardless of the identity of the phagocytic cell responsible, the mechanism involved is likely PS-dependent. Mice lacking ELMO-1, an intracellular adaptor protein required for the transduction of signals from certain PS receptors, had a neurogenic phenotype that mimicked that observed following annexin V treatment (Lu et al., 2011). Still, the exact receptors and ligands mediating phagocytosis in adult (homeostatic) neurogenesis remain elusive and further investigations must be conducted.

## Sertoli cells and spermatogenesis

Male germ cell production, spermatogenesis, is a carefully regulated process involving the differentiation of stem cells, the spermatogonia, to spermatozoa (Shaha et al., 2010). Spermatogenesis is characterized by a balance between cell differentiation and proliferation on one hand, and apoptosis on the other (Russell et al., 2002). It has been estimated that about 75% of developing spermatogonia undergo apoptosis (Huckins, 1978). Such regulated cell death is critical: inhibition of spermatogonia apoptosis results in increased cell death and testicular atrophy (Russell et al., 2002).

The extensive occurrence of cell death is accompanied by rapid phagocytic removal of the apoptotic cells by the Sertoli cells, somatic cells that support the maturation of spermatogonia and maintain homeostasis of the testicular environment (Griswold, 1998; Nakanishi and Shiratsuchi, 2004). The phagocytic activity of Sertoli cells, observed both *in vitro* in primary testicular cultures, and *in vivo*, was determined to be dependent on PS exposure on the surface of apoptotic spermatogenic cells (Shiratsuchi et al., 1997; Kawasaki et al., 2002; Nakagawa et al., 2005). Scavenger receptor B1 was shown to significantly contribute to the engulfment of these developing cells, as inhibitors of this receptor substantially inhibit the clearance of apoptotic spermatogenic cells *in vivo* (Kawasaki et al., 2002; Nakagawa et al., 2005). It is interesting to note that Sertoli cells are non-professional phagocytes, as they are epithelial in nature, with different embryological origins from macrophages (Barrionuevo et al., 2011). The central role of Sertoli cells in spermatogenesis substantiates and emphasizes the requirement for non-professional phagocytes in homeostasis and development.

## Phagocytosis of microbes

As mentioned in the Introduction, phagocytic cells can be divided into professional and non-professional phagocytes (Rabinovitch, 1995). In this section, we will review how the process whereby microbes are engulfed varies among the different types of phagocytic cells.

Neutrophils are the most efficient phagocytes: a single neutrophil can engulf up to 50 bacteria. Furthermore, phagocytosis by neutrophils is very fast, often requiring only a few seconds (Segal et al., 1980). Neutrophils are not only fast and efficient, but also relatively abundant, constituting 50–60% of all leukocytes in human blood. They are also the first cells to be recruited from the blood stream to an infected site (Mayer-Scholl et al., 2004). As neutrophils are circulating cells, they first need to leave the bloodstream and trans-migrate across the endothelium by a process termed diapedesis in order to reach the site of infection. Chemoattractants, secreted by the microorganisms or by the host cells at the site of infection guide the extravasation of neutrophils. Once neutrophils arrive at sites of infection, phagocytosis of the pathogen ensues.

**Figure 3:**
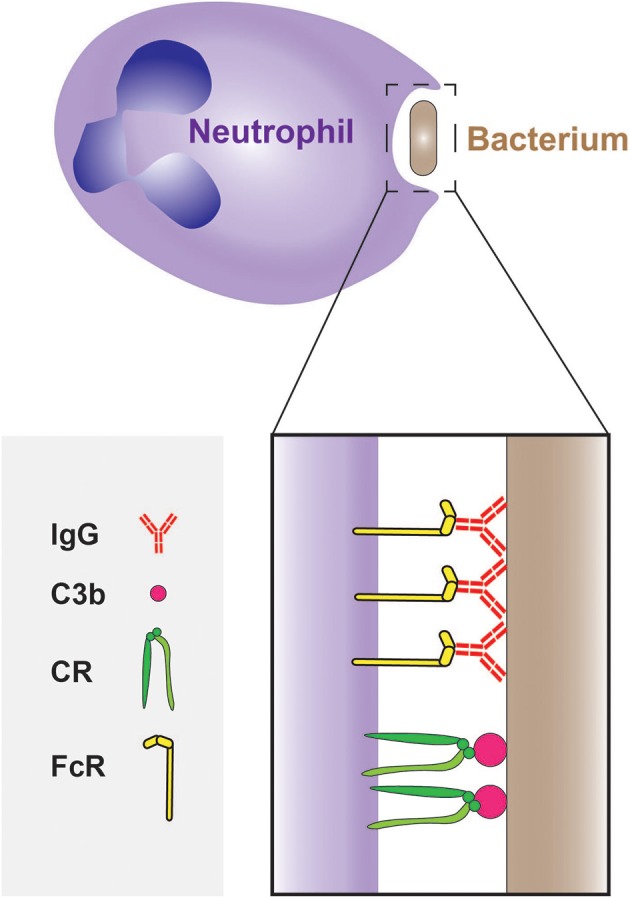
**Phagocytosis of bacteria by neutrophils**. The phagocytosis of bacteria is often mediated by opsonisation of their surface with IgG and C3b molecules, which are recognized by Fcγ receptors (FcγRs) and complement receptors (CRs), respectively.

Neutrophils express receptors that recognize phagocytic determinants that are intrinsic to the pathogens (i.e., PAMPs). These are typified by the C-type lectins Dectin-1–which binds β-glucan–and Dectin-2–which can bind a wide range of ligands on the surface of fungi, mycobacteria and even cancer cells (Kerscher et al., 2013; Kimura et al., 2016). While recognition of PAMPs can trigger phagocytosis, microbial engulfment is optimal when the targets are opsonized, i.e., coated with serum components that are recognized by effective phagocytic receptors. The most abundant opsonins in serum are immunoglobulins and certain components of the complement cascade; these are recognized by Fc receptors (FcRs) and by complement receptors (CRs), respectively (Figure 3). Circulating (quiescent) neutrophils express FcγRII (CD32) and FcγRIII (CD16), but only low levels of FcγRI (CD64) (Hoffmann, 2009), which is instead found on monocytes and macrophages; neutrophils also express FcαR, the IgA receptor. In addition, they express the complement receptors CR1(CD35) and CR3 (CD11b/CD18 or MAC-1) that bind the opsonic molecule C3b (and iC3b).

Interestingly, the opsonic pathways can function synergistically: IgG molecules bound to the pathogen can trigger the complement cascade via the classical pathway, resulting in secondary opsonization of the target with C3b and thereby accelerating the phagocytic process (van Kessel et al., 2014). In contrast, IgA does not activate the complement system.

There are other receptors expressed by neutrophils that are not phagocytic *per se*, but facilitate and enhance phagocytosis, a process known as priming. Priming occurs when the phagocytes are exposed to agents, such as lipopolysaccharide (LPS), TNF-α, or GM-CSF (Aida and Pabst, 1990; Khwaja et al., 1992) prior to or at the time when they encounter their targets. LPS and several other microbial components capable of priming are recognized by Toll-like receptors (TLRs). Of note, neutrophils express most of the known TLR's, including TLR2 that binds lipoteichoic acid of gram-positive bacteria, and TLR4 that binds LPS of gram-negative bacteria (Prince et al., 2011). Priming can enhance expression of phagocytic receptors, such as FcγRI. Indeed, increased surface expression of FcγRI is often used as a marker for bacterial infection (Hoffmann, 2009).

Phagocytosis by neutrophils culminates with the elimination of the ingested microorganisms. The cytoplasmic granules that are characteristic of neutrophils (a type of granulocyte) are instrumental in the microbicidal response. Neutrophils contain both cytoplasmic granules and smaller secretory vesicles that fuse with nascent and maturing phagosomes. The granules are roughly divided into three types: primary (or azurophilic) granules, secondary (or specific) granules, and tertiary (or gelatinase) granules. The granule types are not completely distinct from one another, and there are intermediate species. The azurophilic granules contain a variety of antimicrobial substances, such as assorted lytic enzymes, antimicrobial peptides that include the defensins, and myeloperoxidase, an enzyme that catalyzes the production of hypochlorous acid. The secondary granules contain phagocytic receptors (e.g., FcγRs and CRs) and also the NADPH oxidase complex that produces reactive oxygen species (ROS). The tertiary granules contain receptors and enzymes that degrade extracellular matrix to facilitate the extravasation and migration of the neutrophils to the site of inflammation (Faurschou and Borregaard, 2003; Kolaczkowska and Kubes, 2013).

The second wave of leukocytes to reach the infected site consists of monocytes, which differentiate into macrophages and DCs. There are three subpopulations of monocytes: classical, non-classical and intermediate. The classical monocytes constitute 80–90% of the total blood monocytes; they are virtually all circulating cells. The other 10–20% is comprised of the non-classical or patrolling monocytes, and the intermediate monocytes. The fraction of non-classical cells is probably an underestimate, since the patrolling monocytes are adhered to the endothelium and hence difficult to quantify accurately. The monocyte sub-populations are identified by their relative abundance of two markers: CD14 (an LPS co-receptor) and CD16 (FcγRIII). The classical monocytes have high levels of CD14 and virtually no expression of CD16 (CD14^++^, CD16^−^); non-classical monocytes express lower levels of CD14 and high levels of CD16 (CD14^+^, CD16^++^), and the intermediate monocytes express high levels of CD14 and low levels of CD16 (CD14^++^, CD16^+^). Despite the fact that patrolling monocytes adhere to the vascular endothelium and are hence closer to the tissues where infections generally occur, the classical monocytes are the first to arrive at the infected site. There is no information regarding the ability of the different subpopulations of monocytes to perform phagocytosis *in vivo*, but the limited data obtained *in vitro* suggest that both classical and intermediate monocytes can phagocytose actively, whereas non-classical monocyte are less effective (Mukherjee et al., 2015; Zhou et al., 2015).

Monocyte-derived macrophages express a vast array of phagocytic receptors (as reviewed by Freeman and Grinstein, 2014), such as FcγRs and CRs for opsonized targets, scavenger receptors and C-type lectins, (e.g., mannose receptor and dectins) for fungi. As described for monocytes, not all macrophages are alike; they differ according to the nature of their precursors and of the signals the monocyte/macrophage encounters *en route* and at the site of infection. Most simply, the macrophage can acquire an anti-inflammatory phenotype (termed M2) or a pro-inflammatory phenotype (termed M1). These different phenotypes are associated with distinct arrays of phagocytic receptors; for example, the anti-inflammatory macrophages express more efferocytic receptors than the inflammatory macrophages and can thereby clean and resolve the aftermath of a neutrophil attack. Conversely, inflammatory macrophages express more FcγRs and can therefore ingest IgG-opsonized targets more effectively (Levin et al., 2016). The properties of the phagosomes also differ in the two main types of macrophages: while the phagosomes of anti-inflammatory macrophages acidify quickly (Canton et al., 2014), those of pro-inflammatory macrophages remain neutral and can even become alkaline, resembling the behavior of phagosomes in neutrophils. In the two latter instances, failure to acidify the phagosome is attributed to the massive generation of superoxides that in turn consume protons during the dismutation reaction.

An interplay between monocytes and neutrophils has been observed during fungal infection. The C-type lectin Mincle has reciprocal expression in circulating monocytes and neutrophils: when Mincle is expressed in neutrophils, it is absent in monocytes and vice versa, and this expression pattern has been suggested to have physiological implications (Vijayan et al., 2012). Neutrophils that express Mincle are able to ingest and kill *Candida albicans*, whereas monocytes displaying Mincle do not effectively ingest or kill the fungi, but instead elicit an inflammatory response.

As mentioned earlier, neutrophils, monocytes and monocyte-derived macrophages are attracted to sites of infection, moving up a gradient of attractant signals. However, the true first responders are the phagocytes that reside in the tissues that become infected, i.e., the tissue macrophages. Tissue macrophages are tuned to the environment in which they reside through exposure to tissue-specific factors that affect their morphology and function. It was originally believed that tissue macrophages differentiated from hematopoietic stem cell-derived monocytes. Recently, however, Gomez et al. (Gomez Perdiguero et al., 2015) showed that embryo yolk sac macrophages are a sufficient source for the generation of macrophages in the liver, skin and the central nervous system. Another study showed that fetal monocytes are the precursors of alveolar macrophages (AM), which colonize the airways a few days after birth (Guilliams et al., 2013). Furthermore, it is now apparent that AMs are capable of self-renewal without requiring involvement of monocyte-derived macrophages, even after depletion of alveolar macrophage due to influenza infection (Hashimoto et al., 2013). Fetal monocytes require GM-CSF—which is secreted by the alveolar epithelium—to differentiate into AMs. Accordingly, AMs in GM-CSF-deficient mice are not fully differentiated and are functionally limited; as predicted, addition of exogenous GM-CSF restored their development (Guilliams et al., 2013). Alveolar macrophages seem to have a phenotype that is intermediate between the canonical M1 and M2 polarization states. This is reflected by their unique pattern of expression of markers: transcriptomic analysis showed that they express CD69, TLR2 and 4, CXC-chemokine ligand (CXCL) 9,10 and 11, and CC-chemokine ligand 5 (CCL5), characteristic of M1 cells, but also CD206, MARCO, matrix metalloproteinase (MMP) 2, 7 and 9, the tyrosine protein kinase MER, growth arrest-specific protein 7, CD163, stabilin 1, arginase and the adenosine A3 receptor, that are typical of M2 cells (Shaykhiev et al., 2009). There are additional receptors found on AMs, such as CD11c (Guth et al., 2009; Zaynagetdinov et al., 2013), which is found also in macrophages of the gut mucosa, the lectin Siglec-F that binds sialic acid and is found predominantly on eosinophils (Feng and Mao, 2012; Misharin et al., 2013) Even though (or perhaps because?) they are continuously exposed to inhaled microorganisms and other particulates, AMs show lower phagocytic ability when compared to lung interstitial macrophages (Hussell and Bell, 2014). An *in vivo* study showed that in mice infected with *Streptococcus pneumoniae* neutrophils were recruited to the lungs where they engulfed the bacteria, while the main role of AMs was to resolve the inflammation by clearing the apoptotic neutrophils (Knapp et al., 2003). These findings suggest that the hybrid M1/M2 phenotype of AMs is required to, on one hand, effect an inflammatory response to recruit neutrophils, while serving to resolve the inflammation by engulfing apoptotic neutrophils on the other.

## Phagocytosis of tumor cells

**Figure 4:**
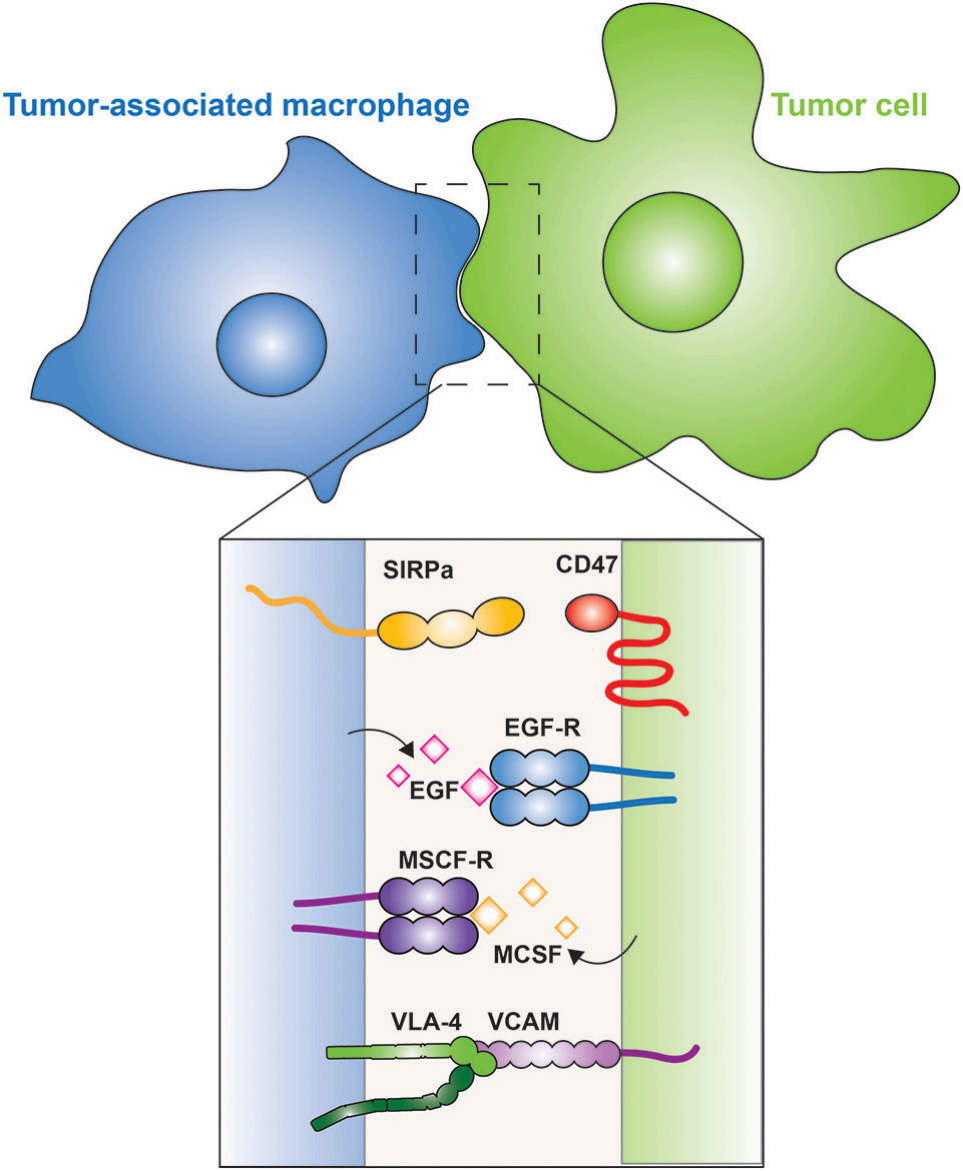
**Tumor-associated macrophages**. The interaction between a macrophage (blue) and a tumor cell (green) is illustrated. The inset shows the membrane proteins involved in tumor cell evasion and promotion. CD47 is a “don't eat me” signal expressed on the tumor cell, which is recognized by SIRPα on the macrophage. Tumor cells secrete M-CSF, which is recognized by the M-CSF receptor on the surface of macrophages, leading to the secretion of EGF by the latter; a positive feedback loop between the two cells is thus generated. Metastatic cells express VCAM that interacts with the integrin VLA-4, which activates cell survival pathways in the metastatic cell.

The response of phagocytes to malignant cells can be ambivalent: M1 macrophages are associated with tumor suppression (Yuan et al., 2015), while neutrophils, classical monocytes and tumor associated-macrophages (TAMs), which are largely polarized toward the M2 phenotype, promote tumorigenesis and aid in immune system evasion (Chanmee et al., 2014; Figure 4).

Recruitment of TAMs to tumors can be mediated by M-CSF, one of the factors that drives the polarization of M2 macrophages. M-CSF is produced and secreted by the tumor cells, which can also secrete other M2-promoting factors, such as IL-10 and CCL-2. The TAMs in turn, secrete epidermal growth factor (EGF) that promotes angiogenesis and induces secretion of more M-CSF by the tumor cells, thus generating a positive feedback loop (Goswami et al., 2005; Hernandez et al., 2009; Figure 4). In metastatic cells, vascular cell adhesion molecule-1 (VCAM-1) is over-expressed and interacts with macrophage integrin α4β1, also known as very late antigen-4 (VLA-4) (Figure 4). This interaction activates the PI3K/Akt pathway in the metastatic cell, promoting its survival in a leukocyte-rich environment (Chen et al., 2011).

In sharp contrast to the tumor- and metastasis-promoting effects of TAMs and other phagocytes, M1 macrophages and, curiously, also patrolling monocytes, have anti-tumor effects (Hanna et al., 2015). Hanna et al. showed that patrolling monocytes in the lung reduce tumor metastasis and recruit natural killer cells. The tumoricidal activity of these phagocytes reflects the balance between “eat me” and “don't eat me” signals. Tumor cells express the “eat me” signal calreticulin on their plasma membrane. Calreticulin is an endoplasmic reticulum chaperone protein that participates in the folding and quality control of *N*-glycosylated proteins. When it escapes the reticulum and translocates to the plasma membrane, calreticulin serves as a phagocytic ligand, i.e., an “eat me” signal. The message of such ligands can be suppressed by “don't eat me” signals, such as those provided by CD47, a ligand for SIRPα, an ITIM-bearing receptor that inhibits phagocytosis. Chao et al. (2010) showed that blocking CD47 on tumor cells while activating calreticulin translocation leads to phagocytosis of the tumor cells. In apoptotic cells calreticulin translocation to the membrane serves as an “eat me” ligand that is recognized by CD91 (LRP-1) on the macrophage, which triggers efferocytosis (Gardai et al., 2005). Clearly, further studies are needed to understand the interaction between macrophages and cancer cells.

## Antigen presentation

**Figure 5:**
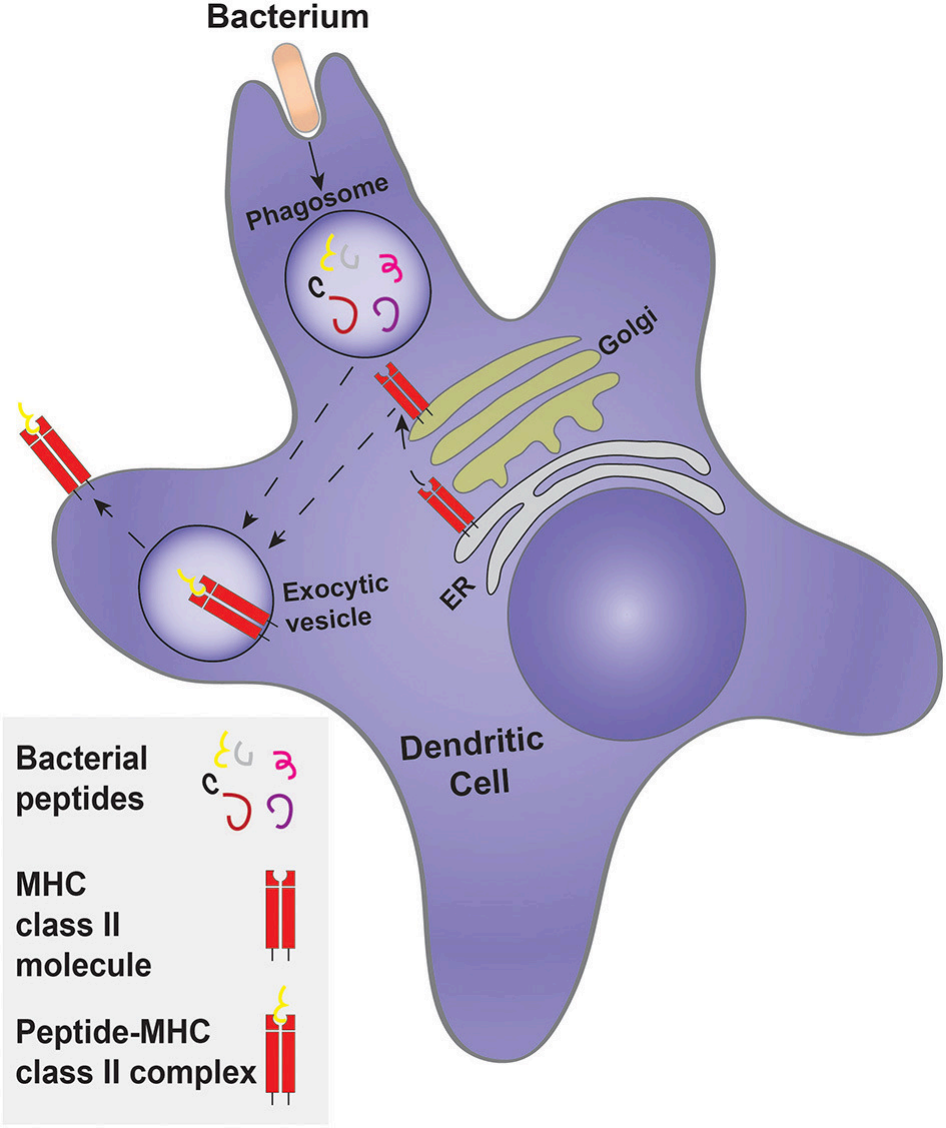
**Classical antigen presentation by a dendritic cell**. Dendritic cells engulf a target, such as bacteria, forming a phagosome. Controlled bacterial degradation in the phagosome generates peptides that are used for antigen presentation on MHC class II molecules. The MHC class II molecule is synthesized and processed by the ER and Golgi complex, being delivered to an exocytosis-competent vesicle where it encounters and is loaded with antigenic peptides to form the peptide-MHC class II complex.

In addition to their role in innate immunity by elimination of pathogens, cell debris and apoptotic cells, phagocytes also participate in the adaptive-immune response by presenting antigens to lymphocytes. Phagocytosis is an important event in antigen presentation. DCs are professional antigen-presenting cells (APCs), whereas neutrophils, macrophages and even epithelial cells can present antigens, but with considerably less efficiency. DCs are derived from hematopoietic stem cells of the bone marrow, and are divided into three subpopulations: conventional DCs (cDCs), plasmacytoid DCs (pDCs) and monocyte-derived DCs (moDCs). The differentiation of monocytes into moDCs occurs at sites of infection (Schraml and Reis e Sousa, 2015). The unique ability of DCs to present antigens is due, at least in part, to the manner whereby their phagosomes mature. Like other professional phagocytes, DCs engulf pathogens but instead of completely destroying them—as macrophages and neutrophils tend to do—DC phagosomes perform controlled proteolysis, which favors the generation of peptides suitable for binding by major histocompatibility complex molecules (MHC), that ultimately present the antigens to lymphoid cells at the plasma membrane (Savina and Amigorena, 2007). Antigens that are engulfed and degraded in the phagosome are loaded to class II MHC molecules (Cresswell, 1994; Figure 5). In some instances, such antigens can reach the cytosol and are then translocated into the endoplasmic reticulum, where they associate with MHC class I molecules for eventual delivery to the cell surface, a process termed cross-presentation (Joffre et al., 2012).

Prior to their activation, DCs are termed immature and express a large variety of phagocytic receptors, such as FcRs and scavenger receptors, as well as TLRs (Guermonprez et al., 2002). The phagocytic receptors expressed by the three sub-populations of DCs are not identical. For example, the moDCs express both activating and inhibitory FcRs, whereas the cDCs and moDCs express a higher proportion of inhibitory FcRs (Guilliams et al., 2014). Phagosome formation and maturation in DCs is controlled and tailored for antigen presentation. While neutrophils and macrophages are mainly concerned with eliminating the pathogen, DCs exert mild proteolysis to generate appropriately sized peptides that can be used for presentation. Indeed, Nagl et al. (2002), showed that macrophages ingest and kill *E. coli* and *S. aureus* more efficiently than DCs. Accordingly, bone marrow-derived DCs have lower levels of proteases than macrophages (Delamarre et al., 2005; Savina et al., 2006). In addition, the phagosomes of immature DCs do not acidify. This has been attributed to the partial assembly of the V-ATPase, the main lysosomal proton transporter, and/or to the heightened activity of the NADPH oxidase 2 (NOX2), that generates copious amounts of superoxide; dismutation of the latter to hydrogen peroxide consumes protons, elevating the luminal pH of the phagosome. The more alkaline pH leads to lower proteolytic activity, which favors generation of peptides suitable for antigen presentation (Figure 5).

Phagocytosis triggers a drastic phenotypic change in the DCs. This so-called maturation process involves substantial morphological and functional changes. The antigen uptake associated with phagocytosis—and to a lesser extent by with macropinocytosis—is followed by a virtual loss in macropinocytic activity and a marked reduction phagocytic capacity (Garrett et al., 2000); notably, receptor-mediated endocytosis persists in the mature cells (Platt et al., 2010). The molecular basis of the down-regulation of phagocytosis is poorly understood. The changes undergone by the mature DC minimize the acquisition of additional antigens, while optimizing the presentation of those acquired by the immature cell. Mature DCs migrate to the lymph node, where they present antigens and activate the adaptive immune system (Guermonprez et al., 2002).

## Concluding remarks

Since Metchnikoff discovered phagocytosis in the context of innate immunity, the functional roles of the phenomenon have multiplied. The importance of phagocytosis in tissue homeostasis and remodeling is now widely appreciated, as is its role in coupling innate and adaptive immunity via antigen presentation. These more recent discoveries have unmasked the existence of myriad receptors, signal transduction pathways and membrane trafficking routes that are only partially understood. The molecular basis of the functional versatility of phagocytes and of phagocytosis is only now beginning to be unraveled. We anticipate the discovery of additional signals associated with phagosome formation and maturation that will be conveyed to other organelles and may direct transcription of genes that will in turn inform surrounding cells and tissues of the metabolic status of the phagocytes. These speculative ideas are amenable to experimental testing, which we hope will be the subject of future investigation.

## Author contributions

JL, SG, and ZR conceived and wrote the review.

## Funding

Supported by grant FDN143202 from the Canadian Institutes of Health Research.

### Conflict of interest statement

The authors declare that the research was conducted in the absence of any commercial or financial relationships that could be construed as a potential conflict of interest. The reviewer YA and handling Editor declared their shared affiliation, and the handling Editor states that the process nevertheless met the standards of a fair and objective review.

## References

[B1] Aida Y. Pabst M. J. (1990). Priming of neutrophils by lipopolysaccharide for enhanced release of superoxide. Requirement for plasma but not for tumor necrosis factor-alpha. J. Immunol. 145, 3017–3025. 2170529

[B2] Antonelou M. H. Kriebardis A. G. Papassideri I. S. (2010). Aging and death signalling in mature red cells: from basic science to transfusion practice. Blood Transfus. 8, 39–47. 10.2450/2010.007S 20606748 PMC2897187

[B3] Arandjelovic S. Ravichandran K. S. (2015). Phagocytosis of apoptotic cells in homeostasis. Nat. Immunol. 16, 907–917. 10.1038/ni.3253 26287597 PMC4826466

[B4] Arese P. Turrini F. Schwarzer E. (2005). Band 3/complement-mediated recognition and removal of normally senescent and pathological human erythrocytes. Cell. Physiol. Biochem. 16, 133–146. 10.1159/000089839 16301814

[B5] Awasaki T. Ito K. (2004). Engulfing action of glial cells is required for programmed axon pruning during *Drosophila* metamorphosis. Curr. Biol. 14, 668–677. 10.1016/j.cub.2004.04.001 15084281

[B6] Barrionuevo F. Burgos M. Jiménez R. (2011). Origin and function of embryonic Sertoli cells. Biomol. Concepts 2, 537–547. 10.1515/BMC.2011.044 25962053

[B7] Boas F. E. Forman L. Beutler E. (1998). Phosphatidylserine exposure and red cell viability in red cell aging and in hemolytic anemia. Proc. Natl. Acad. Sci. U.S.A. 95, 3077–3081. 10.1073/pnas.95.6.3077 9501218 PMC19697

[B8] Braun S. M. G. Jessberger S. (2014). Adult neurogenesis: mechanisms and functional significance. Development 141, 1983–1986. 10.1242/dev.104596 24803647

[B9] Brown G. C. Neher J. J. (2012). Eaten alive! Cell death by primary phagocytosis: “phagoptosis.” Trends Biochem. Sci. 37, 325–332. 10.1016/j.tibs.2012.05.002 22682109

[B10] Butterfield T. A. Best T. M. Merrick M. A. (2006). The dual roles of neutrophils and macrophages in inflammation: a critical balance between tissue damage and repair. J. Athl. Train. 41, 457–465. 10.1016/S0162-0908(08)79217-1 17273473 PMC1748424

[B11] Campbell F. R. (1968). Nuclear elimination from the normoblast of fetal guinea pig liver as studied with electron microscopy and serial sectioning techniques. Anat. Rec. 160, 539–554. 10.1002/ar.1091600304 4874448

[B12] Canton J. Khezri R. Glogauer M. Grinstein S. (2014). Contrasting phagosome pH regulation and maturation in human M1 and M2 macrophages. Mol. Biol. Cell 25, 3330–3341. 10.1091/mbc.E14-05-0967 25165138 PMC4214780

[B13] Chanmee T. Ontong P. Konno K. Itano N. (2014). Tumor-associated macrophages as major players in the tumor microenvironment. Cancers (Basel). 6, 1670–1690. 10.3390/cancers6031670 25125485 PMC4190561

[B14] Chao M. P. Jaiswal S. Weissman-Tsukamoto R. Alizadeh A. A. Gentles A. J. Volkmer J.. (2010). Calreticulin is the dominant pro-phagocytic signal on multiple human cancers and is counterbalanced by CD47. Sci. Transl. Med. 2, 63ra94. 10.1126/scitranslmed.3001375 21178137 PMC4126904

[B15] Chen Q. Zhang X. H.-F. Massagué J. (2011). Macrophage binding to receptor VCAM-1 transmits survival signals in breast cancer cells that invade the lungs. Cancer Cell 20, 538–549. 10.1016/j.ccr.2011.08.025 22014578 PMC3293160

[B16] Connor J. Pak C. C. Schroit A. J. (1994). Exposure of phosphatidylserine in the outer leaflet of human red blood cells: relationship to cell density, cell age, and clearance by mononuclear cells. J. Biol. Chem. 269, 2399–2404. 8300565

[B17] Cox G. Crossley J. Xing Z. (1995). Macrophage engulfment of apoptotic neutrophils contributes to the resolution of acute pulmonary inflammation *in vivo*. Am. J. Respir. Cell Mol. Biol. 12, 232–237. 10.1165/ajrcmb.12.2.7865221 7865221

[B18] Crehan H. Hardy J. Pocock J. (2013). Blockage of CR1 prevents activation of rodent microglia. Neurobiol. Dis. 54, 139–149. 10.1016/j.nbd.2013.02.003 23454195

[B19] Cresswell P. (1994). Assembly, transport, and function of MHC class II molecules. Annu. Rev. Immunol. 12, 259–293. 10.1146/annurev.iy.12.040194.001355 8011283

[B20] de Back D. Z. Kostova E. B. van Kraaij M. van den Berg T. K. van Bruggen R. (2014). Of macrophages and red blood cells; a complex love story. Front. Physiol. 5:9. 10.3389/fphys.2014.00009 24523696 PMC3906564

[B21] Delamarre L. Pack M. Chang H. Mellman I. Trombetta E. S. (2005). Differential lysosomal proteolysis in antigen-presenting cells determines antigen fate. Science 307, 1630–1634. 10.1126/science.1108003 15761154

[B22] Doherty J. Logan M. A. Taademir O. E. Freeman M. R. (2009). Ensheathing glia function as phagocytes in the adult *Drosophila* brain. J. Neurosci. 29, 4768–4781. 10.1523/JNEUROSCI.5951-08.2009 19369546 PMC2674269

[B23] Dzierzak E. Philipsen S. (2013). Erythropoiesis: development and differentiation. Cold Spring Harb. Perspect. Med. 3:a011601. 10.1101/cshperspect.a011601 23545573 PMC3684002

[B24] Fadok V. A. Warner M. L. Bratton D. L. Henson P. M. (1998). CD36 is required for phagocytosis of apoptotic cells by human macrophages that use either a phosphatidylserine receptor or the vitronectin receptor (α_v_β_3_). J. Immunol. 161, 6250–6257. 9834113

[B25] Faurschou M. Borregaard N. (2003). Neutrophil granules and secretory vesicles in inflammation. Microbes Infect. 5, 1317–1327. 10.1016/j.micinf.2003.09.008 14613775

[B26] Feng Y. Mao H. (2012). Expression and preliminary functional analysis of Siglec-F on mouse macrophages. J. Zhejiang Univ. Sci. B 13, 386–394. 10.1631/jzus.B1100218 22556177 PMC3348230

[B27] Freeman S. A. Grinstein S. (2014). Phagocytosis: receptors, signal integration, and the cytoskeleton. Immunol. Rev. 262, 193–215. 10.1111/imr.12212 25319336

[B28] Gabillet J. Millet A. Pederzoli-Ribeil M. Tacnet-Delorme P. Guillevin L. Mouthon L.. (2012). Proteinase 3, the autoantigen in granulomatosis with polyangiitis, associates with calreticulin on apoptotic neutrophils, impairs macrophage phagocytosis, and promotes inflammation. J. Immunol. 189, 2574–2583. 10.4049/jimmunol.1200600 22844112

[B29] Gardai S. J. McPhillips K. A. Frasch S. C. Janssen W. J. Starefeldt A. Murphy-Ullrich J. E.. (2005). Cell-surface calreticulin initiates clearance of viable or apoptotic cells through trans-activation of LRP on the phagocyte. Cell 123, 321–334. 10.1016/j.cell.2005.08.032 16239148

[B30] Garrett W. S. Chen L. M. Kroschewski R. Ebersold M. Turley S. Trombetta S.. (2000). Developmental control of endocytosis in dendritic cells by Cdc42. Cell 102, 325–334. 10.1016/S0092-8674(00)00038-6 10975523

[B31] Gattegno L. Saffar L. Vaysse J. (1989). Inhibition by monoclonal anticomplement receptor type 1 on interactions between senescent human red blood cells and monocytic-macrophagic cells. J. Leukoc. Biol. 45, 422–428. 2708912 10.1002/jlb.45.5.422

[B32] Gayen Betal S. Setty B. N. Y. (2008). Phosphatidylserine-positive erythrocytes bind to immobilized and soluble thrombospondin-1 via its heparin-binding domain. Transl. Res. 152, 165–177. 10.1016/j.trsl.2008.07.007 18940719 PMC2628802

[B33] Gomez Perdiguero E. Klapproth K. Schulz C. Busch K. Azzoni E. Crozet L.. (2015). Tissue-resident macrophages originate from yolk-sac-derived erythro-myeloid progenitors. Nature 518, 547–551. 10.1038/nature13989 25470051 PMC5997177

[B34] Gordon S. (2008). Elie metchnikoff: father of natural immunity. Eur. J. Immunol. 38, 3257–3264. 10.1002/eji.200838855 19039772

[B35] Gordon S. (2016). Phagocytosis: an Immunobiologic Process. Immunity 44, 463–475. 10.1016/j.immuni.2016.02.026 26982354

[B36] Goswami S. Sahai E. Wyckoff J. B. Cammer M. Cox D. Pixley F. J.. (2005). Macrophages promote the invasion of breast carcinoma cells via a colony-stimulating factor-1/epidermal growth factor paracrine loop. Cancer Res. 65, 5278–5283. 10.1158/0008-5472.CAN-04-1853 15958574

[B37] Griswold M. D. (1998). The central role of Sertoli cells in spermatogenesis. Semin. Cell Dev. Biol. 9, 411–416. 10.1006/scdb.1998.0203 9813187

[B38] Guermonprez P. Valladeau J. Zitvogel L. Théry C. Amigorena S. (2002). Antigen presentation and T cell stimulation by dendritic cells. Annu. Rev. Immunol. 20, 621–667. 10.1146/annurev.immunol.20.100301.064828 11861614

[B39] Guilliams M. Bruhns P. Saeys Y. Hammad H. Lambrecht B. N. (2014). The function of Fcγ receptors in dendritic cells and macrophages. Nat. Rev. Immunol. 14, 94–108. 10.1038/nri3582 24445665

[B40] Guilliams M. De Kleer I. Henri S. Post S. Vanhoutte L. De Prijck S.. (2013). Alveolar macrophages develop from fetal monocytes that differentiate into long-lived cells in the first week of life via GM-CSF. J. Exp. Med. 210, 1977–1992. 10.1084/jem.20131199 24043763 PMC3782041

[B41] Guth A. M. Janssen W. J. Bosio C. M. Crouch E. C. Henson P. M. Dow S. W. (2009). Lung environment determines unique phenotype of alveolar macrophages. Am. J. Physiol. Lung Cell. Mol. Physiol. 296, L936–L946. 10.1152/ajplung.90625.2008 19304907 PMC2692811

[B42] Hanna R. N. Cekic C. Sag D. Tacke R. Thomas G. D. Nowyhed H.. (2015). Patrolling monocytes control tumor metastasis to the lung. Science 350, 985–990. 10.1126/science.aac9407 26494174 PMC4869713

[B43] Hashimoto D. Chow A. Noizat C. Teo P. Beasley M. B. Leboeuf M.. (2013). Tissue-resident macrophages self-maintain locally throughout adult life with minimal contribution from circulating monocytes. Immunity 38, 792–804. 10.1016/j.immuni.2013.04.004 23601688 PMC3853406

[B44] Haslett C. (1992). Resolution of acute inflammation and the role of apoptosis in the tissue fate of granulocytes. Clin. Sci. 83, 639–648. 10.1042/cs0830639 1336433

[B45] Haslett C. Savill J. S. Whyte M. K. Stern M. Dransfield I. Meagher L. C. (1994). Granulocyte apoptosis and the control of inflammation. Philos. Trans. R. Soc. Lond. B Biol. Sci. 345, 327–333. 10.1098/rstb.1994.0113 7846130

[B46] Hentze M. W. Muckenthaler M. U. Galy B. Camaschella C. (2010). Two to tango: regulation of mammalian iron metabolism. Cell 142, 24–38. 10.1016/j.cell.2010.06.028 20603012

[B47] Hernandez L. Smirnova T. Kedrin D. Wyckoff J. Zhu L. Stanley E. R.. (2009). The EGF/CSF-1 paracrine invasion loop can be triggered by heregulin beta1 and CXCL12. Cancer Res. 69, 3221–3227. 10.1158/0008-5472.CAN-08-2871 19293185 PMC2820720

[B48] Hochreiter-Hufford A. Ravichandran K. S. (2013). Clearing the dead: apoptotic cell sensing, recognition, engulfment, and digestion. Cold Spring Harb. Perspect. Biol. 5:a008748. 10.1101/cshperspect.a008748 23284042 PMC3579390

[B49] Hoffmann J. J. M. L. (2009). Neutrophil CD64 : a diagnostic marker for infection and sepsis. Clin. Chem. Lab. Med. 47, 903–916. 10.1515/cclm.2009.224 19642859

[B50] Huckins C. (1978). The morphology and kinetics of spermatogonial degeneration in normal adult rats: an analysis using a simplified classification of the germinal epithelium. Anat. Rec. 190, 905–926. 10.1002/ar.1091900410 637327

[B51] Hussell T. Bell T. J. (2014). Alveolar macrophages: plasticity in a tissue-specific context. Nat. Rev. Immunol. 14, 81–93. 10.1038/nri3600 24445666

[B52] Huttenlocher P. R. Dabholkar A. S. (1997). Regional differences in synaptogenesis in human cerebral cortex. J. Comp. Neurol. 387, 167–178. 10.1002/(sici)1096-9861(19971020)387:2<167::aid-cne1>3.0.co;2-z 9336221

[B53] Ishii Y. Hashimoto K. Nomura A. Sakamoto T. Uchida Y. Ohtsuka M.. (1998). Elimination of neutrophils by apoptosis during the resolution of acute pulmonary inflammation in rats. Lung 176, 89–98. 10.1007/PL00007597 9500294

[B54] Joffre O. P. Segura E. Savina A. Amigorena S. (2012). Cross-presentation by dendritic cells. Nat. Rev. Immunol. 12, 557–569. 10.1038/nri3254 22790179

[B55] Kannan R. Yuan J. Low P. S. (1991). Isolation and partial characterization of antibody- and globin-enriched complexes from membranes of dense human erythrocytes. Biochem. J. 278 (Pt 1), 57–62. 10.1042/bj2780057 1883341 PMC1151448

[B56] Kawane K. Fukuyama H. Kondoh G. Takeda J. Ohsawa Y. Uchiyama Y.. (2001). Requirement of DNase II for definitive erythropoiesis in the mouse fetal liver. Science 292, 1546–1549. 10.1126/science.292.5521.1546 11375492

[B57] Kawasaki Y. Nakagawa A. Nagaosa K. Shiratsuchi A. Nakanishi Y. (2002). Phosphatidylserine binding of class B scavenger receptor type I, a phagocytosis receptor of testicular sertoli cells. J. Biol. Chem. 277, 27559–27566. 10.1074/jbc.M202879200 12016218

[B58] Kerscher B. Willment J. A. Brown G. D. (2013). The Dectin-2 family of C-type lectin-like receptors: an update. Int. Immunol. 25, 271–277. 10.1093/intimm/dxt006 23606632 PMC3631001

[B59] Kettenmann H. Hanisch U.-K. Noda M. Verkhratsky A. (2011). Physiology of microglia. Physiol. Rev. 91, 461–553. 10.1152/physrev.00011.2010 21527731

[B60] Khandelwal S. Van Rooijen N. Saxena R. K. (2007). Reduced expression of CD47 during murine red blood cell (RBC) senescence and its role in RBC clearance from the circulation. Transfusion 47, 1725–1732. 10.1111/j.1537-2995.2007.01348.x 17725740

[B61] Khwaja A. Carver J. E. Linch D. C. (1992). Interactions of granulocyte-macrophage colony-stimulating factor (CSF), granulocyte CSF, and tumor necrosis factor alpha in the priming of the neutrophil respiratory burst. Blood 79, 745–753. 1370644

[B62] Kimura Y. Inoue A. Hangai S. Saijo S. Negishi H. Nishio J.. (2016). The innate immune receptor Dectin-2 mediates the phagocytosis of cancer cells by Kupffer cells for the suppression of liver metastasis. Proc. Natl. Acad. Sci. U.S.A. 113, 14097–14102. 10.1073/pnas.1617903113 27872290 PMC5150405

[B63] Kinchen J. M. Ravichandran K. S. (2008). Phagosome maturation: going through the acid test. Nat. Rev. Mol. Cell Biol. 9, 781–795. 10.1038/nrm2515 18813294 PMC2908392

[B64] Knapp S. Leemans J. C. Florquin S. Branger J. Maris N. A. Pater J.. (2003). Alveolar macrophages have a protective antiinflammatory role during murine pneumococcal pneumonia. Am. J. Respir. Crit. Care Med. 167, 171–179. 10.1164/rccm.200207-698OC 12406830

[B65] Koh T. J. DiPietro L. A. (2011). Inflammation and wound healing: the role of the macrophage. Expert Rev. Mol. Med. 13:e23. 10.1017/S1462399411001943 21740602 PMC3596046

[B66] Kolaczkowska E. Kubes P. (2013). Neutrophil recruitment and function in health and inflammation. Nat. Rev. Immunol. 13, 159–175. 10.1038/nri3399 23435331

[B67] Korotzer A. R. Watt J. Cribbs D. Tenner A. J. Burdick D. Glabe C.. (1995). Cultured rat microglia express C1q and receptor for C1q: implications for amyloid effects on microglia. Exp. Neurol. 134, 214–221. 10.1006/exnr.1995.1051 7556541

[B68] Lee S. J. Park S. Y. Jung M. Y. Bae S. M. Kim I. S. (2011). Mechanism for phosphatidylserine-dependent erythrophagocytosis in mouse liver. Blood 117, 5215–5223. 10.1182/blood-2010-10-313239 21427291

[B69] Levin R. Grinstein S. Canton J. (2016). The life cycle of phagosomes: formation, maturation, and resolution. Immunol. Rev. 273, 156–179. 10.1111/imr.12439 27558334

[B70] Lin Z. Schmidt C. Q. Koutsogiannaki S. Ricci P. Risitano A. M. Lambris J. D.. (2015). Complement C3dg-mediated erythrophagocytosis: implications for paroxysmal nocturnal hemoglobinuria. Blood 126, 891–894. 10.1182/blood-2015-02-625871 26082452 PMC4536542

[B71] London A. Cohen M. Schwartz M. (2013). Microglia and monocyte-derived macrophages: functionally distinct populations that act in concert in CNS plasticity and repair. Front. Cell. Neurosci. 7:34. 10.3389/fncel.2013.00034 23596391 PMC3625831

[B72] Low L. K. Cheng H.-J. (2006). Axon pruning: an essential step underlying the developmental plasticity of neuronal connections. Philos. Trans. R. Soc. Lond. B Biol. Sci. 361, 1531–1544. 10.1098/rstb.2006.1883 16939973 PMC1664669

[B73] Lu Z. Elliott M. R. Chen Y. Walsh J. T. Klibanov A. L. Ravichandran K. S.. (2011). Phagocytic activity of neuronal progenitors regulates adult neurogenesis. Nat. Cell Biol. 13, 1076–1083. 10.1038/ncb2299 21804544 PMC3374401

[B74] Luo C. Koyama R. Ikegaya Y. (2016). Microglia engulf viable newborn cells in the epileptic dentate gyrus. Glia 64, 1508–1517. 10.1002/glia.23018 27301702

[B75] Lutz H. U. Bussolino F. Flepp R. Fasler S. Stammler P. Kazatchkine M. D.. (1987). Naturally occurring anti-band-3 antibodies and complement together mediate phagocytosis of oxidatively stressed human erythrocytes. Proc. Natl. Acad. Sci. U.S.A. 84, 7368–7372. 10.1073/pnas.84.21.7368 3313392 PMC299297

[B76] Lutz H. U. Stammler P. Fasler S. (1990). How naturally occurring anti-band 3 antibodies stimulate C3b deposition to senescent and oxidatively stressed red blood cells. Biomed. Biochim. Acta 49, S224–S229. 2386510

[B77] Mallat M. Marín-Teva J. L. Chéret C. (2005). Phagocytosis in the developing CNS: more than clearing the corpses. Curr. Opin. Neurobiol. 15, 101–107. 10.1016/j.conb.2005.01.006 15721751

[B78] Manwani D. Bieker J. J. (2008). The Erythroblastic island. Curr. Top. Dev. Biol. 82, 23–53. 10.1016/S0070-2153(07)00002-6 18282516 PMC3234703

[B79] Mayer-Scholl A. Averhoff P. Zychlinsky A. (2004). How do neutrophils and pathogens interact? Curr. Opin. Microbiol. 7, 62–66. 10.1016/j.mib.2003.12.004 15036142

[B80] McEvoy L. Williamson P. Schlegel R. A. (1986). Membrane phospholipid asymmetry as a determinant of erythrocyte recognition by macrophages. Proc. Natl. Acad. Sci. U.S.A. 83, 3311–3315. 10.1073/pnas.83.10.3311 3458184 PMC323503

[B81] Misharin A. V. Morales-Nebreda L. Mutlu G. M. Budinger G. R. S. Perlman H. (2013). Flow cytometric analysis of macrophages and dendritic cell subsets in the mouse lung. Am. J. Respir. Cell Mol. Biol. 49, 503–510. 10.1165/rcmb.2013-0086MA 23672262 PMC3824047

[B82] Mohandas N. Prenant M. (1978). Three-dimensional model of bone marrow. Blood 51, 633–643. 630113

[B83] Mukherjee R. Kanti Barman P. Kumar Thatoi P. Tripathy R. Kumar Das B. Ravindran B. (2015). Non-classical monocytes display inflammatory features: validation in sepsis and systemic lupus erythematous. Sci. Rep. 5, 138–186. 10.1038/srep13886 26358827 PMC4566081

[B84] Nagl M. Kacani L. Müllauer B. Lemberger E.-M. Stoiber H. Sprinzl G. M.. (2002). Phagocytosis and killing of bacteria by professional phagocytes and dendritic cells. Clin. Diagn. Lab. Immunol. 9, 1165–1168. 10.1128/cdli.9.6.1165-1168.2002 12414745 PMC130096

[B85] Nakagawa A. Shiratsuchi A. Tsuda K. Nakanishi Y. (2005). *In vivo* analysis of phagocytosis of apoptotic cells by testicular Sertoli cells. Mol. Reprod. Dev. 71, 166–177. 10.1002/mrd.20278 15791597

[B86] Nakanishi Y. Shiratsuchi A. (2004). Phagocytic removal of apoptotic spermatogenic cells by Sertoli cells: mechanisms and consequences. Biol. Pharm. Bull. 27, 13–16. 10.1248/bpb.27.13 14709891

[B87] Olsson M. Oldenborg P. A. (2008). CD47 on experimentally senescent murine RBCs inhibits phagocytosis following Fcγ receptor-mediated but not scavenger receptor-mediated recognition by macrophages. Blood 112, 4259–4267. 10.1182/blood-2008-03-143008 18779391

[B88] Paolicelli R. C. Bolasco G. Pagani F. Maggi L. Scianni M. Panzanelli P.. (2011). Synaptic pruning by microglia is necessary for normal brain development. Science 333, 1456–1458. 10.1126/science.1202529 21778362

[B89] Petanjek Z. Judas M. Simic G. Rasin M. R. Uylings H. B. M. Rakic P.. (2011). Extraordinary neoteny of synaptic spines in the human prefrontal cortex. Proc. Natl. Acad. Sci. U.S.A. 108, 13281–13286. 10.1073/pnas.1105108108 21788513 PMC3156171

[B90] Platt C. D. Ma J. K. Chalouni C. Ebersold M. Bou-Reslan H. Carano R. A. D.. (2010). Mature dendritic cells use endocytic receptors to capture and present antigens. Proc. Natl. Acad. Sci. U.S.A. 107, 4287–4292. 10.1073/pnas.0910609107 20142498 PMC2840134

[B91] Potter P. K. Cortes-Hernandez J. Quartier P. Botto M. Walport M. J. (2003). Lupus-prone mice have an abnormal response to thioglycolate and an impaired clearance of apoptotic cells. J. Immunol. 170, 3223–3232. 10.4049/jimmunol.170.6.3223 12626581

[B92] Prince L. R. Whyte M. K. Sabroe I. Parker L. C. (2011). The role of TLRs in neutrophil activation. Curr. Opin. Pharmacol. 11, 397–403. 10.1016/j.coph.2011.06.007 21741310

[B93] Psaila B. Barkas N. Iskander D. Roy A. Anderson S. Ashley N.. (2016). Single-cell profiling of human megakaryocyte-erythroid progenitors identifies distinct megakaryocyte and erythroid differentiation pathways. Genome Biol. 17:83. 10.1186/s13059-016-0939-7 27142433 PMC4855892

[B94] Qiu L. B. Dickson H. Hajibagheri N. Crocker P. R. (1995). Extruded erythroblast nuclei are bound and phagocytosed by a novel macrophage receptor. Blood 85, 1630–1639. 7888682

[B95] Rabinovitch M. (1995). Professional and non-professional phagocytes: an introduction. Trends Cell Biol. 5, 85–87. 10.1016/S0962-8924(00)88955-2 14732160

[B96] Russell L. D. Chiarini-Garcia H. Korsmeyer S. J. Knudson C. M. (2002). Bax-dependent spermatogonia apoptosis is required for testicular development and spermatogenesis. Biol. Reprod. 66, 950–958. 10.1095/biolreprod66.4.950 11906913

[B97] Sambrano G. R. Parthasarathy S. Steinberg D. (1994). Recognition of oxidatively damaged erythrocytes by a macrophage receptor with specificity for oxidized low density lipoprotein. Proc. Natl. Acad. Sci. U.S.A. 91, 3265–3269. 10.1073/pnas.91.8.3265 8159736 PMC43557

[B98] Sasaki K. Iwatsuki H. Suda M. Itano C. (1993a). Cell death and phagocytosis of haematopoietic elements at the onset of haematopoiesis in the mouse spleen: an ultrastructural study. J. Anat. 183(Pt 1), 113–20. 8270466 PMC1259859

[B99] Sasaki K. Iwatsuki H. Suda M. Itano C. (1993b). Scavenger macrophages and central macrophages of erythroblastic islands in liver hemopoiesis of the fetal and early postnatal mouse: a semithin light-and electron-microscopic study. Acta Anat. (Basel). 147, 75–82. 10.1159/000147485 8379295

[B100] Savill J. Dransfield I. Hogg N. Haslett C. (1990). Vitronectin receptor-mediated phagocytosis of cells undergoing apoptosis. Nature 343, 170–173. 10.1038/343170a0 1688647

[B101] Savill J. Hogg N. Ren Y. Haslett C. (1992). Thrombospondin cooperates with CD36 and the vitronectin receptor in macrophage recognition of neutrophils undergoing apoptosis. J. Clin. Invest. 90, 1513–1522. 10.1172/JCI116019 1383273 PMC443198

[B102] Savill J. S. Wyllie A. H. Henson J. E. Walport M. J. Henson P. M. Haslett C. (1989). Macrophage phagocytosis of aging neutrophils in inflammation. Programmed cell death in the neutrophil leads to its recognition by macrophages. J. Clin. Invest. 83, 865–875. 10.1172/JCI113970 2921324 PMC303760

[B103] Savina A. Amigorena S. (2007). Phagocytosis and antigen presentation in dendritic cells. Immunol. Rev. 219, 143–156. 10.1111/j.1600-065X.2007.00552.x 17850487

[B104] Savina A. Jancic C. Hugues S. Guermonprez P. Vargas P. Moura I. C.. (2006). NOX2 controls phagosomal pH to regulate antigen processing during crosspresentation by dendritic cells. Cell 126, 205–218. 10.1016/j.cell.2006.05.035 16839887

[B105] Schafer D. P. Lehrman E. K. Kautzman A. G. Koyama R. Mardinly A. R. Yamasaki R.. (2012). Microglia sculpt postnatal neural circuits in an activity and complement-dependent manner. Neuron 74, 691–705. 10.1016/j.neuron.2012.03.026 22632727 PMC3528177

[B106] Schraml B. U. Reis e Sousa C. (2015). Defining dendritic cells. Curr. Opin. Immunol. 32, 13–20. 10.1016/j.coi.2014.11.001 25553392

[B107] Segal A. W. Dorling J. Coade S. (1980). Kinetics of fusion of the cytoplasmic granules with phagocytic vacuoles in human polymorphonuclear leukocytes. Biochemical and morphological studies. J. Cell Biol. 85, 42–59. 10.1083/jcb.85.1.42 7364874 PMC2110597

[B108] Shaha C. Tripathi R. Mishra D. P. (2010). Male germ cell apoptosis: regulation and biology. Philos. Trans. R. Soc. Lond. B Biol. Sci. 365, 1501–1515. 10.1098/rstb.2009.0124 20403866 PMC2871916

[B109] Shaykhiev R. Krause A. Salit J. Strulovici-Barel Y. Harvey B.-G. O'Connor T. P.. (2009). Smoking-dependent reprogramming of alveolar macrophage polarization: implication for pathogenesis of chronic obstructive pulmonary disease. J. Immunol. 183, 2867–2883. 10.4049/jimmunol.0900473 19635926 PMC2873685

[B110] Shiratsuchi A. Umeda M. Ohba Y. Nakanishi Y. (1997). Recognition of phosphatidylserine on the surface of apoptotic spermatogenic cells and subsequent phagocytosis by sertoli cells of the rat. J. Biol. Chem. 272, 2354–2358. 10.1074/jbc.272.4.2354 8999945

[B111] Sierra A. Encinas J. M. Deudero J. J. P. Chancey J. H. Enikolopov G. Overstreet-Wadiche L. S.. (2010). Microglia shape adult hippocampal neurogenesis through apoptosis-coupled phagocytosis. Cell Stem Cell 7, 483–495. 10.1016/j.stem.2010.08.014 20887954 PMC4008496

[B112] Skutelsky E. Danon D. (1969). Reduction in surface charge as an explanation of the recognition by macrophages of nuclei expelled from normoblasts. J. Cell Biol. 43, 8–15. 10.1083/jcb.43.1.8 4186414 PMC2107843

[B113] Stevens B. Allen N. J. Vazquez L. E. Howell G. R. Christopherson K. S. Nouri N.. (2007). The classical complement cascade mediates CNS synapse elimination. Cell 131, 1164–1178. 10.1016/j.cell.2007.10.036 18083105

[B114] Tang G. Gudsnuk K. Kuo S. H. Cotrina M. L. Rosoklija G. Sosunov A.. (2014). Loss of mTOR-dependent macroautophagy causes autistic-like synaptic pruning deficits. Neuron 83, 1131–1143. 10.1016/j.neuron.2014.07.040 25155956 PMC4159743

[B115] Terpstra V. van Berkel T. J. C. (2000). Scavenger receptors on liver Kupffer cells mediate the *in vivo* uptake of oxidatively damaged red blood cells in mice. Blood 95, 2157–2163. 10.1073/pnas.83.5.1339 10706889

[B116] Theurl I. Hilgendorf I. Nairz M. Tymoszuk P. Haschka D. Asshoff M.. (2016). On-demand erythrocyte disposal and iron recycling requires transient macrophages in the liver. Nat. Med. 22, 945–951. 10.1038/nm.4146 27428900 PMC4957133

[B117] Tollis S. Dart A. E. Tzircotis G. Endres R. G. (2010). The zipper mechanism in phagocytosis: energetic requirements and variability in phagocytic cup shape. BMC Syst. Biol. 4:149. 10.1186/1752-0509-4-149 21059234 PMC2991294

[B118] Torre D. Gennero L. Baccino F. M. Speranza F. Biondi G. Pugliese A. (2002). Impaired macrophage phagocytosis of apoptotic neutrophils in patients with human immunodeficiency virus type 1 infection. Clin. Diagn. Lab. Immunol. 9, 983–986. 10.1128/cdli.9.5.983-986.2002 12204947 PMC120074

[B119] van Kessel K. P. M. Bestebroer J. van Strijp J. A. G. (2014). Neutrophil-Mediated Phagocytosis of Staphylococcus aureus. Front. Immunol. 5:467. 10.3389/fimmu.2014.00467 25309547 PMC4176147

[B120] Vijayan D. Radford K. J. Beckhouse A. G. Ashman R. B. Wells C. A. (2012). Mincle polarizes human monocyte and neutrophil responses to *Candida albicans*. Immunol. Cell Biol. 90, 889–895. 10.1038/icb.2012.24 22641025

[B121] Visser C. E. Steenbergen J. J. Betjes M. G. Meijer S. Arisz L. Hoefsmit E. C.. (1995). Interleukin-8 production by human mesothelial cells after direct stimulation with staphylococci. Infect. Immun. 63, 4206–4209. 7558346 10.1128/iai.63.10.4206-4209.1995 PMC173597

[B122] Wake H. Moorhouse A. J. Jinno S. Kohsaka S. Nabekura J. (2009). Resting microglia directly monitor the functional state of synapses *in vivo* and determine the fate of ischemic terminals. J. Neurosci. 29, 3974–3980. 10.1523/JNEUROSCI.4363-08.2009 19339593 PMC6665392

[B123] Watts R. J. Schuldiner O. Perrino J. Larsen C. Luo L. (2004). Glia engulf degenerating axons during developmental axon pruning. Curr. Biol. 14, 678–684. 10.1016/j.cub.2004.03.035 15084282

[B124] Wynn T. A. Vannella K. M. (2016). Macrophages in tissue repair, regeneration, and fibrosis. Immunity 44, 450–462. 10.1016/j.immuni.2016.02.015 26982353 PMC4794754

[B125] Yoshida H. Kawane K. Koike M. Mori Y. Uchiyama Y. Nagata S. (2005). Phosphatidylserine-dependent engulfment by macrophages of nuclei from erythroid precursor cells. Nature 437, 754–758. 10.1038/nature03964 16193055

[B126] Yuan A. Hsiao Y.-J. Chen H.-Y. Chen H.-W. Ho C.-C. Chen Y.-Y.. (2015). Opposite effects of M1 and M2 macrophage subtypes on lung cancer progression. Sci. Rep. 5:14273. 10.1038/srep14273 26399191 PMC4585843

[B127] Zaynagetdinov R. Sherrill T. P. Kendall P. L. Segal B. H. Weller K. P. Tighe R. M.. (2013). Identification of myeloid cell subsets in murine lungs using flow cytometry. Am. J. Respir. Cell Mol. Biol. 49, 180–189. 10.1165/rcmb.2012-0366MA 23492192 PMC3824033

[B128] Zhou J. Feng G. Beeson J. Hogarth P. M. Rogerson S. J. Yan Y. (2015). CD14^hi^CD16+ monocytes phagocytose antibody-opsonised Plasmodium falciparum infected erythrocytes more efficiently than other monocyte subsets, and require CD16 and complement to do so. BMC Med. 13:154 10.1186/s12916-015-0391-7 26149666 PMC4493812

